# Effect of balanced crystalloids versus saline on urinary biomarkers of acute kidney injury in critically ill adults

**DOI:** 10.1186/s12882-021-02236-x

**Published:** 2021-02-05

**Authors:** Blake E. Funke, Karen E. Jackson, Wesley H. Self, Sean P. Collins, Christina T. Saunders, Li Wang, Jeffrey D. Blume, Nancy Wickersham, Ryan M. Brown, Jonathan D. Casey, Gordon R. Bernard, Todd W. Rice, Edward D. Siew, Matthew W. Semler, for the SMART Investigators, Gordon R. Bernard, Ryan M. Brown, Jonathan D. Casey, Todd W. Rice, Matthew W. Semler, Christopher J. Lindsell, Li Wang, Jonathan P. Wanderer, Wesley H. Self, Edward D. Siew, Joanna L. Stollings, the Pragmatic Critical Care Research Group

**Affiliations:** 1 Department of Medicine, Vanderbilt University Medical Center, Nashville, TN USA; 2 Division of Allergy, Pulmonary, and Critical Care Medicine, Vanderbilt University Medical Center, C-1216 MCN, 1161 21st Ave South, Nashville, TN 37232 USA; 3 Department of Emergency Medicine, Vanderbilt University Medical Center, Nashville, TN USA; 4 Department of Biostatistics, Vanderbilt University Medical Center, Nashville, TN USA; 5 Division of Nephrology and Hypertension, Vanderbilt Center for Kidney Disease (VCKD) and Integrated Program for AKI (VIP-AKI), Vanderbilt University Medical Center, Nashville, TN USA

**Keywords:** Critical care, Resuscitation, Renal insufficiency, Dialysis, Sodium chloride, Sepsis

## Abstract

**Background:**

Recent trials have suggested use of balanced crystalloids may decrease the incidence of major adverse kidney events compared to saline in critically ill adults. The effect of crystalloid composition on biomarkers of early acute kidney injury remains unknown.

**Methods:**

From February 15 to July 15, 2016, we conducted an ancillary study to the Isotonic Solutions and Major Adverse Renal Events Trial (SMART) comparing the effect of balanced crystalloids versus saline on urinary levels of neutrophil gelatinase-associated lipocalin (NGAL) and kidney injury molecule-1 (KIM-1) among 261 consecutively-enrolled critically ill adults admitted from the emergency department to the medical ICU. After informed consent, we collected urine 36 ± 12 h after hospital admission and measured NGAL and KIM-1 levels using commercially available ELISAs. Levels of NGAL and KIM-1 at 36 ± 12 h were compared between patients assigned to balanced crystalloids versus saline using a Mann-Whitney U test.

**Results:**

The 131 patients (50.2%) assigned to the balanced crystalloid group and the 130 patients (49.8%) assigned to the saline group were similar at baseline. Urinary NGAL levels were significantly lower in the balanced crystalloid group (median, 39.4 ng/mg [IQR 9.9 to 133.2]) compared with the saline group (median, 64.4 ng/mg [IQR 27.6 to 339.9]) (*P* < 0.001). Urinary KIM-1 levels did not significantly differ between the balanced crystalloid group (median, 2.7 ng/mg [IQR 1.5 to 4.9]) and the saline group (median, 2.4 ng/mg [IQR 1.3 to 5.0]) (*P* = 0.36).

**Conclusions:**

In this ancillary analysis of a clinical trial comparing balanced crystalloids to saline among critically ill adults, balanced crystalloids were associated with lower urinary concentrations of NGAL and similar urinary concentrations of KIM-1, compared with saline. These results suggest only a modest reduction in early biomarkers of acute kidney injury with use of balanced crystalloids compared with saline.

**Trial registration:**

ClinicalTrials.gov number: NCT02444988. Date registered: May 15, 2015.

**Supplementary Information:**

The online version contains supplementary material available at 10.1186/s12882-021-02236-x.

## Background

Administration of intravenous fluid is common in critical care [1]. Historically, 0.9% sodium chloride (saline) has been the most frequently administered fluid [2]. Balanced crystalloid solutions, such as lactated Ringer’s solution and Plasma-Lyte, represent an increasingly used alternative to saline [3]. Recent data from clinical trials suggest that crystalloid composition may affect patient outcomes [4], including kidney injury, but the mechanistic effects of balanced crystalloids versus saline on the development of acute kidney injury (AKI) remain uncertain.

Pre-clinical studies have found the chloride content of intravenous fluids influences renal vasoconstriction [5], glomerular filtration rate [5], biomarkers of AKI [6], and incidence of AKI by clinical criteria and histology [7]. Randomized trials among healthy human volunteers suggest that intravenous solutions with high chloride content, such as 0.9% saline, may decrease renal artery blood velocity, renal cortical perfusion, and urine output [8–10]. A small randomized trial among patients undergoing major abdominal surgery found lower concentrations of urinary neutrophil gelatinase-associated lipocalin (NGAL), an early biomarker of renal tubular injury, with balanced crystalloids compared to saline [11]. Observational studies have reported lower rates of AKI [12] and renal replacement therapy (RRT) [13] among acutely ill adults treated with balanced crystalloids rather than saline. Two recent clinical trials found balanced crystalloid solutions decreased the composite of death, RRT, or persistent renal dysfunction during acute illness [4, 14]. Although plasma creatinine concentration did not differ between the balanced crystalloids and saline in these trials, serum creatinine is an insensitive marker for tubular injury. Whether the differences in clinical outcomes between balanced crystalloids and saline in these trials were mediated by kidney injury or by other mechanisms remains unknown.

NGAL and kidney injury molecule-1 (KIM-1) are glycoproteins produced in renal tubular epithelial cells in response to renal tubular injury. Through different mechanisms, both proteins appear to provide renal protection in response to tubular injury and both have been established as sensitive early urinary biomarkers of tubular injury [15–17]. NGAL functions as an iron-binding protein and a growth factor and is synthesized in both proximal and distal tubule cells. Its renal protective effect is thought to be due to its role in iron scavenging and delivery or its ability to induce cell growth and differentiation [15]. In the kidney, KIM-1 is expressed only in proximal tubule cells and is thought to facilitate remodeling of injured epithelia via clearance of apoptotic and necrotic cells.

To better understand the effect of crystalloid composition on the development of tubular injury, we conducted an ancillary study comparing urinary levels of the proximal and distal tubule biomarker NGAL and proximal tubule biomarker KIM-1 between critically ill adults assigned to balanced crystalloids versus saline as part of a large pragmatic trial [4]. We hypothesized that levels of these urinary biomarkers would be lower among patients assigned to balanced crystalloids compared with patients assigned to saline.

## Methods

### Study design and oversight

To examine the effects of balanced crystalloids versus saline on early biomarkers of acute tubular injury, we performed a pre-specified ancillary study within a large, pragmatic trial comparing balanced crystalloids to saline among critically ill adults. This study adheres to CONSORT guidelines.

The Isotonic Solutions and Major Adverse Renal Events Trial (SMART) was a cluster-randomized, cluster-crossover trial comparing balanced crystalloids to saline for intravenous fluid administration among 15,802 critically ill adults admitted to five intensive care units (ICUs) at Vanderbilt University Medical Center between June 1, 2015 and April 30, 2017 [4]. The SMART trial was approved by the Institutional Review Board at Vanderbilt University (IRB #141349). Details of the design, analysis, and results of SMART have been published previously [4, 18].

The Vanderbilt University Emergency Medicine Biomarkers Study is an ongoing repository of biological samples collected from patients treated for acute illness in the Adult Emergency Department at Vanderbilt University Medical Center. This observational cohort study was approved by the Institutional Review Board at Vanderbilt University (IRB #111510). Patients or their legally authorized representatives provided written informed consent before enrollment.

The cohort for the current study included all patients co-enrolled in SMART and the Vanderbilt University Emergency Medicine Biomarkers Study between February 15, 2016 and July 15, 2016.

### Patient population

From February 15, 2016 through July 15, 2016, study personnel for the Vanderbilt University Emergency Medicine Biomarkers Study screened consecutive adults admitted from the emergency department (ED) to the medical ICU. Adult (age ≥ 18 years) patients admitted from the ED, alive, and physically located in the medical ICU between 24 and 48 h after hospital admission were eligible.

### Treatment assignment and intervention

Per the SMART protocol, for each month of the trial, participating ICUs were assigned to use either balanced crystalloids (the treating clinician’s choice of lactated Ringer’s solution or Plasma-Lyte A®) or saline (0.9% sodium chloride) for any intravenous isotonic crystalloid administration (Figure S1). Saline contains 154 mmol per liter of sodium and chloride, whereas both lactated Ringer’s solution and Plasma-Lyte A® contain lower concentrations of chloride, small amount of potassium, and buffering anions such as lactate, acetate, or gluconate (Table S1). Fluid administration in the ED was coordinated with the fluid assigned to the medical ICU. Patients, clinicians, and investigators were not blinded to group assignment.

### Collection of biospecimens

After obtaining informed consent, study personnel collected 5 mL of urine from the patient or the patient’s urinary catheter. Urine was collected as close to 36 h after hospital admission as feasible, and not less than 24 h or more than 48 h after hospital admission. The period of 24–48 h after hospital admission is referred to as “day 2” throughout this manuscript. Study personnel then screened urine samples being held in the clinical lab at 4° Celsius from the first 6 h of the patient’s initial ED presentation. When samples were available, the study team collected 5 mL of urine. Samples from the first 6 h of the patient’s initial ED presentation are referred to as “day 0” samples. All urine was centrifuged at 1000 g’s for 10 min and frozen at − 80 °C.

### Urinary biomarker measurement

NGAL (also referred to as lipocalin-2 or oncogene 24p3) and KIM-1 (also referred to as hepatitis A virus cellular receptor 1 or T-cell immunoglobulin and mucin domain 1) biomarkers were measured in duplicate in thawed urine by a commercially available Solid Phase Sandwich ELISA (R & D Systems, Minneapolis, MN). The coefficient of variation for each biomarker measurement was less than 10% (Table S2). Background on the normal range and kinetics for NGAL and KIM-1 is provided in the Supplemental Methods.

### Data collection

In addition to biomarker concentrations, study personnel blinded to study group assignment collected clinical data from the electronic health record. Data included pre-enrollment renal function, demographics, orders for intravenous fluids, plasma creatinine values, and clinical outcomes.

### Study outcomes

The co-primary outcomes for the current study were urinary concentrations (scaled to urinary creatinine) of NGAL and KIM-1 on day 2.

Secondary biomarker outcomes included the change in NGAL and KIM-1 concentration (scaled to urinary creatinine concentration) from day 0 to day 2 among patients with urine available from day 0. Secondary clinical outcomes included 30-day in-hospital mortality, stage II or greater acute kidney injury (AKI) according to Kidney Disease: Improving Global Outcomes (KDIGO) creatinine criteria [19], highest creatinine level before discharge or day 30, final creatinine value before discharge or day 30, new receipt of renal-replacement therapy (RRT), the number of days alive and free from the ICU, mechanical ventilation, vasopressor use and RRT, and the proportion of patients who met one or more criteria for a major adverse kidney event within 30 days (MAKE30) – the composite of death, new receipt of RRT, or persistent renal dysfunction [4, 20].

### Statistical analysis

The sample size for the current study was determined by the fixed number of patients co-enrolled in SMART and the Vanderbilt University Emergency Medicine Biomarkers Study between February 15, 2016 and July 15, 2016. Although a prospective sample size calculation was not performed, we estimated that the number of patients co-enrolled would be similar to the 270 patients enrolled in a recent secondary analysis of a randomized trial evaluating the effect of fluid management strategy on urinary NGAL and KIM-1 [21].

Analyses were conducted at the level of each patient’s hospitalization in an intention-to-treat fashion. Continuous variables are reported as mean ± SD or median and interquartile range (IQR); categorical variables are reported as frequencies and proportions.

The primary analysis compared urinary KIM-1 and NGAL levels on day 2 between the balanced crystalloid and saline groups using a Mann-Whitney U test.

We performed multiple secondary analyses. We compared secondary outcomes between groups using the Mann-Whitney U test for continuous variables and the Chi-square test for categorical variables. To evaluate for heterogeneity of treatment effect (subgroup analysis), we fit proportional odds models with the co-primary outcomes as the dependent variables and independent variables of study group, pre-specified potential effect modifiers, and the interaction between the two (see Supplemental Methods). To increase statistical power by controlling for variation and to account for any chance imbalances between study groups in this subset of trial participants, we compared the co-primary outcomes between study groups in proportional odds models adjusting for age, sex, race, receipt of mechanical ventilation, receipt of vasopressors, and diagnosis of sepsis.

A two-sided *P* value < 0.05 indicated statistical significance for the co-primary outcomes. All secondary analyses were considered hypothesis generating and no corrections for multiple testing were performed. All analyses were performed using R version 3.3.0 software (R Foundation for Statistical Computing, Vienna, Austria).

## Results

### Derivation of the study cohort

A total of 261 patients admitted from the ED between February 15, 2016 and July 15, 2016 and located in the medical ICU on day 2 of hospital admission provided informed consent for biospecimen collection and were included in the current analysis (Figure S2). Of these, 131 (50.2%) were assigned to the balanced crystalloid group in SMART and 130 (49.8%) were assigned to the saline group. A total of 111 of the 261 patients (42.5%) also had urine samples available from day 0, of whom 60 (54.1%) were in the balanced crystalloid group and 51 (45.9%) were in the saline group.

### Baseline characteristics

Among the 261 patients enrolled, median age was 58 years, 12% of patients had stage III or greater chronic kidney disease (CKD) (as defined in the supplemental methods), 26% had stage II or greater AKI at ED presentation (prior to the intervention), 26% had a diagnosis of sepsis, and 8% were receiving vasopressors. Patients assigned to the balanced crystalloid group (*n* = 131) and saline group (*n* = 130) were similar at baseline with respect to age, gender, pre-existing chronic kidney disease, and baseline plasma creatinine concentration (Table 1, Tables S3–4). Among the 111 patients with urinary biomarkers measured on day 0, the balanced crystalloid and saline groups did not differ with regard to urinary NGAL level (median, 70 [IQR, 33 to 442] vs 81 [IQR, 28 to 461]; *P* = 0.89) or KIM-1 level (median, 3.5 [IQR, 2.1 to 6.9] vs 3.1 [IQR, 1.4 to 5.5]; *P* = 0.36).

**Table 1:** Patient Characteristics at Baseline

Patient Characteristics^a^	Balanced	Saline
	(***n*** = 131)	(***n*** = 130)
Age – years	55 [43–67]	59 [47–71]
Men – no. (%)	61 (47%)	67 (52%)
Caucasian – no. (%)	112 (85%)	106 (82%)
Weight – kg	82 [68–98]	78 [68–95]
Medical comorbidities – no. (%)		
Hypertension	79 (60%)	69 (53%)
Diabetes	52 (40%)	49 (38%)
Chronic respiratory failure	40 (31%)	39 (30%)
Immunosuppression	30 (23%)	35 (27%)
Coronary artery disease	31 (24%)	24 (18%)
Renal comorbidities – no. (%)		
Chronic kidney disease, stage III or greater^b^	18 (14%)	14 (11%)
Location immediately prior to the intensive care unit – no. (%)		
Emergency department	108 (82%)	105 (81%)
Operating room	2 (2%)	2 (2%)
Hospital ward	21 (16%)	23 (18%)
Admitting diagnosis – no. (%)		
Sepsis or septic shock	36 (27%)	33 (25%)
Respiratory failure	28 (21%)	34 (26%)
Gastrointestinal bleed	16 (12%)	19 (15%)
Mechanical ventilation – no. (%)	17 (13%)	19 (15%)
Vasopressors – no. (%)	10 (8%)	12 (9%)
APACHE II score	11 [7.5–15]	13 [9–16.2]
Baseline creatinine^c^ – mg/dL	0.77 [0.67–0.90]	0.78 [0.65–0.90]
Day 0 NGAL level^e^	70 [33–442]	81 [28–461]
Day 0 KIM-1 level^e^	3.5 [2.1–6.9]	3.1 [1.4–5.5]
Acute kidney injury, stage II or greater^d^	35 (26%)	34 (27%)

^a^Continuous data are presented as median [25th percentile – 75th percentile] unless otherwise noted. Categorical data are presented as number (no.) and percentage (%). There were no significant differences in baseline characteristics between the two study groups (*P* values range from 0.05 to 0.78)

^b^Chronic kidney disease stage III or greater is defined as a glomerular filtration rate less than 60 ml/min per 1.73 m^2^ as calculated by the Chronic Kidney Disease Epidemiology (CKD-EPI) Collaboration equation [22] using the patient’s baseline creatinine value

^c^Baseline creatinine for the study is defined as the lowest plasma creatinine measured in the 12 months prior to hospitalization if available, otherwise the lowest plasma creatinine measured between hospitalization and intensive care unit admission; using an estimated creatinine only for patients without an available plasma creatinine between 12 months prior to hospitalization and the time of ED presentation. A total of 37 patients (28%) in the balanced crystalloid group and 45 patients (35%) in the saline group did not have a measured plasma creatinine value available between 12 months prior to hospital admission and the time of intensive care unit admission

^d^Acute kidney injury, stage II or greater is defined according to Kidney Disease Improving Global Outcomes (KDIGO) creatinine criteria [19] as a first plasma creatinine value at time of ED presentation at least 200% of the baseline value OR both (1) greater than 4.0 mg/dL and (2) increased at least 0.3 mg/dL from the baseline value

^e^Day 0 urine samples were available for 60 patients (46%) in the balanced group and 51 patients (39%) in the saline group

### Fluid therapy and electrolytes

In the ED prior to ICU admission, patients in the balanced crystalloid group received a mean of 991 ± 1189 mL of balanced crystalloids and 307 ± 787 mL of saline, whereas patients in the saline group received a mean of 13 ± 98 mL of balanced crystalloids and 1372 ± 1654 mL of saline (*P* < 0.001) (Table S5). Between ICU admission and day 2, patients in the balanced crystalloid group received a mean of 1911 ± 2277 mL of balanced crystalloid and 153 ± 618 mL of saline, whereas patients in the saline group received a mean of 256 ± 1499 of balanced crystalloid and 1641 ± 1973 mL of saline (*P* < 0.001). The volume of non-study intravenous fluids did not differ between groups (Table S6). Plasma chloride concentrations were lower and plasma bicarbonate concentrations were higher in the balanced crystalloid group compared with the saline group (Table S7).

### Co-primary outcomes

**Fig. 1:**
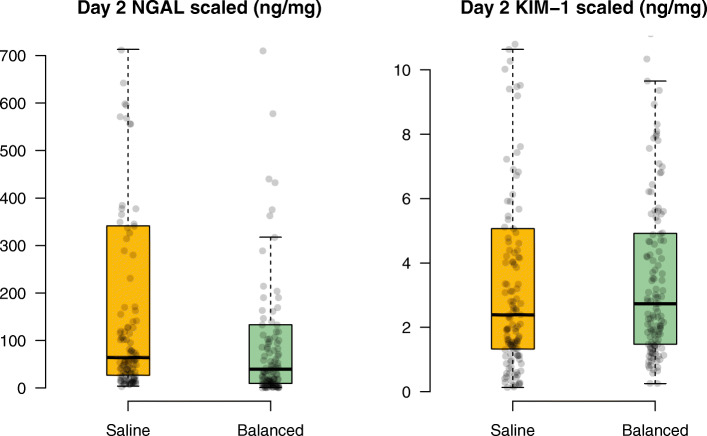
Urinary biomarker levels on day 2 by study group. Urinary NGAL and KIM-1 concentration levels scaled to urinary creatinine concentration are displayed for patients in the balanced crystalloid group (green) and saline group (yellow). From top to bottom, the three horizontal lines on each colored box show the upper quartile, median, and lower quartile, and the vertical dashed lines extend to 1.5 times the interquartile range. Each boxplot is overlaid with on dot for each patient’s observed biomarker values

Among all 261 patients, urinary NGAL levels on day 2 were significantly lower in the balanced crystalloid group (median, 39.4 ng/mg; IQR, 9.9 to 133.2) compared with the saline group (median, 64.4 ng/mg; IQR 27.6 to 339.9) (*P* < 0.001). Urinary KIM-1 levels on day 2 did not differ between the balanced crystalloid group (median, 2.7 ng/mg; IQR, 1.5 to 4.9) and the saline group (median, 2.4 ng/mg; IQR, 1.3 to 5.0) (*P* = 0.36) (Fig. 1). Results were similar in analyses adjusting for pre-specified baseline covariates (Tables S8–9).

**Fig. 2:**
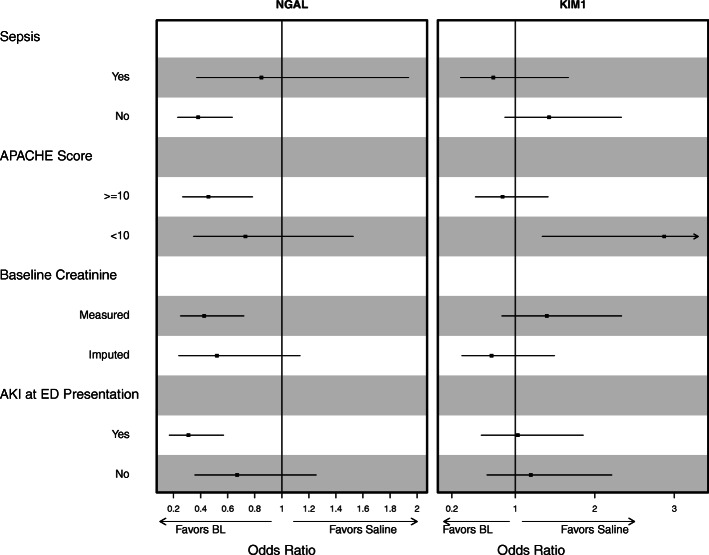
Subgroup analyses. The effects of balanced crystalloids versus saline on day 2 urinary NGAL and KIM-1 concentrations are displayed for patients in each pre-specified subgroup. Scores on the Acute Physiology and Chronic Health Evaluation (APACHE) II range from 0 to 71, with higher scores indicating a greater severity of illness [23]. Acute kidney injury (AKI) of stage 2 or higher is defined according to the Kidney Disease: Improving Global Outcomes creatinine criteria [19]

In subgroup analyses, the effect of balanced crystalloids versus saline on urinary NGAL levels on day 2 appeared to be greater among patients without sepsis, patients with higher APACHE II scores, and patients with AKI on ED presentation (Fig. 2).

### Secondary outcomes

There was no significant difference between groups in secondary clinical outcomes, including stage II or greater AKI, 30-day in-hospital mortality, new receipt of renal replacement therapy, and major adverse kidney events within 30 days (Table 2 and Tables S10–11).

**Table 2:** Clinical Outcomes

Outcome^a^	n	Balanced (***n*** = 131)	Saline (***n*** = 130)	***P*** Value
**Primary Outcomes**				
Day 2 urinary NGAL level – ng/mg (IQR)	261	39.4 [9.9–133.2]	64.4 [27.6–339.9]	< 0.001
Day 2 urinary KIM-1 level – ng/mg (IQR)	261	2.7 [1.5–4.9]	2.4 [1.3–5.0]	0.36
**Secondary Biomarker Outcomes**				
Change in NGAL level from day 0 to 2	111	−27.7 [− 293.4–2.3]	−7.8 [− 115.9–25.7]	0.14
Change in KIM-1 level from day 0 to 2	111	−0.59 [− 2.9–1.21]	−0.29 [− 1.48–0.48]	0.79
**Secondary Clinical Outcomes**				
30-day in-hospital mortality – no. (%)	261	5 (4%)	10 (8%)	–
Major Adverse Kidney Event within 30 days – no. (%)^b^	261	13 (10%)	17 (13%)	–
Intensive care unit-free days^c^	260	25.0 [23.0–26.0]	25.0 [23.0–25.0]	–
Mean ± SD		22.5 ± 6.5	21.8 ± 7.2	
Ventilator-free days^c^	260	28.0 [28.0–28.0]	28.0 [27.0–28.0]	–
Mean ± SD		25.8 ± 6.3	25.1 ± 7.6	
Vasopressor-free days^c^	260	28.0 [27.0–28.0]	28.0 [28.0–28.0]	–
Mean ± SD		26.0 ± 6.2	25.6 ± 7.1	
Renal replacement therapy-free days^c^	260	28.0 [28.0–28.0]	28.0 [28.0–28.0]	–
Mean ± SD		26.6 ± 5.8	25.3 ± 7.9	
Stage II or greater AKI developing after enrollment – no. (%)^d^	261	50 (39%)	48 (37%)	–
New receipt of RRT	261	1 (1%)	3 (2%)	–
Creatinine, mg/dL				
Highest before discharge or day 30	260	1.32 [0.95–1.96]	1.17 [0.81–1.85]	–
Final value before discharge or 30 days	259	0.84 [0.72–1.15]	0.79 [0.68–1.07]	–

^a^Continuous data are presented as median [25th percentile – 75th percentile] unless otherwise noted. Categorical data are presented as number (no.) and percentage (%)

^b^Major Adverse Kidney Events within 30 days (MAKE30) is the composite of death, receipt of new renal replacement therapy, or final creatinine ≥200% baseline, all censored at the first of hospital discharge or 30 days after intensive care unit admission

^c^Intensive care unit-, ventilator-, vasopressor-, and renal replacement therapy-free days refer to the number of days alive and free from the specified therapy in the first 28 days after enrollment

^d^Stage II or greater acute kidney injury (AKI) developing after enrollment is defined using the Kidney Disease Improving Global Outcomes (KDIGO) creatinine criteria [19] as (1) any creatinine value between enrollment and discharge or 30 days that is (a) increased at least 0.3 mg/dL from a preceding post-enrollment value and (b) at least 200% of the baseline value, at least 200% of a preceding post-enrollment value, or at least 4.0 mg/dL; (2) urine output < 0.5 ml/k/h for at least 12 consecutive hours; or (3) new receipt of renal replacement therapy

**Fig. 3:**
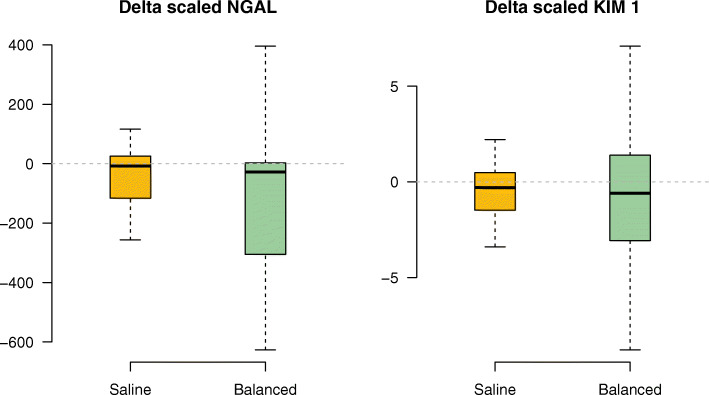
Change in urinary biomarker levels from day 0 to day 2 by study group. The change in urinary NGAL and KIM-1 concentration from emergency department presentation to day 2 of hospital admission scaled to urinary creatinine concentration is displayed for patients in the balanced crystalloid group (green) and saline group (yellow), among the 111 patients with urine available at both time points. From top to bottom, the three horizontal lines on each colored box show the upper quartile, median, and lower quartile, and the vertical dashed lines extend to 1.5 times the interquartile range

Among the 111 patients with urine available from ED presentation, urinary NGAL levels on day 2 appeared to be lower in the balanced crystalloid group (median, 47.4 ng/mg; IQR, 8.4 to 150.6) compared with the saline group (median, 59.4 ng/mg; IQR, 27.9 to 301.7) (*P* = 0.059). Urinary KIM-1 levels on day 2 did not differ between the balanced crystalloid group (median, 2.6 ng/mg; IQR, 1.5 to 5.8) and the saline group (median, 2.6 ng/mg; IQR, 1.5 to 5.0) (*P* = 0.78). The change in urinary NGAL level from day 0 to day 2 was − 27.7 ng/mg (IQR − 293.4 to 2.3) in the balanced crystalloid group versus − 7.8 ng/mg (IQR − 115.9 to 25.7) in the saline group (*P* = 0.14). Change in urinary KIM-1 level from day 0 to day 2 was − 0.59 ng/mg (IQR − 2.9 to 1.21) in the balanced crystalloid group versus − 0.29 ng/mg (IQR − 1.48 to 0.48) in the saline group (*P* = 0.79) (Fig. 3, Figure S3).

## Discussion

In this ancillary analysis of a large clinical trial comparing balanced crystalloids to saline among critically ill adults, balanced crystalloids were associated with modestly lower urinary concentrations of NGAL and similar urinary concentrations of KIM-1 compared with saline. These findings provide the first examination of the effect of crystalloid composition on biomarkers of early AKI in critical illness and inform one of the mechanisms by which crystalloid composition may affect clinical outcomes.

Prior studies have evaluated the effect of balanced crystalloids versus saline on biomarkers of AKI in animal models. Zhou et al. reported that in a rat model of sepsis, resuscitation with a balanced crystalloid solution resulted in lower urinary concentrations of NGAL compared to saline [6]. In this model, the higher NGAL concentrations with saline corresponded with higher stages of AKI and loss of brush border, vacuolization, and dilation of the tubular lumen on renal histology.

Recent studies have examined the effect of crystalloid composition on biomarkers of AKI in humans. In a randomized controlled trial among adult patients undergoing major abdominal surgery, Volta et al. found that intraoperative use of balanced solutions rather than unbalanced solutions resulted in lower urinary NGAL concentrations at the end of surgery, 2 h after surgery, and on post-operative day 1 [11]. Additionally, Dey et al. reported that in patients undergoing elective craniotomy for supratentorial brain tumors, serum NGAL concentrations were significantly lower 3 h after surgery in patients randomized to intra-operative Plasma-Lyte compared to saline [24].

Our findings are consistent with this prior research suggesting that crystalloid composition may influence urinary concentrations of NGAL. High chloride delivery to the distal tubule has been hypothesized to increase tubuloglomerular feedback, decrease renal blood flow, and contribute to ischemic injury to the kidney [5]. These effects might be greater among patients who have already experienced acute kidney injury and might influence recovery from kidney injury instead of or in addition to development of new kidney injury. In the current study, the difference between balanced crystalloids and saline in urinary NGAL concentration was larger among patients with AKI at the time of presentation. This is similar to the finding in prior trials that the effect of balanced crystalloids versus saline on clinical outcomes appeared to be greatest among patients with AKI at the time of presentation [14].

To our knowledge, this is also the first study to compare the effect of balanced crystalloids versus saline crystalloids on urinary KIM-1 levels. Prior work has examined the effect of hypertonic saline on urinary KIM-1. In a study by Mose et al. [25], healthy subjects were given an intravenous load of 3% saline over 60 min accompanied by placebo or furosemide. In subjects randomized to 3% saline and placebo, urinary KIM-1 levels were significantly increased from baseline in the 150–210 min after infusion.

Several potential reasons might explain why we observed differences between balanced crystalloids and saline in urinary NGAL levels but not KIM-1 levels at 36 h. First, differences in the kinetics of expression for the two biomarkers may have contributed to these findings. For example, in patients in which the timing of the kidney insult is known (e.g. cardiac surgery), urinary NGAL levels appear to peak sooner (hours) in patients with AKI compared to similar studies with KIM-1, which tended to peak at 3 days after surgery [26, 27]. Thus, it is possible that any putative effect of crystalloid type on kidney injury biomarker expression may not have yet been fully manifest with urinary KIM-1 at the timepoint we assessed. Differences may also exist in the threshold expression of these biomarkers in response to crystalloid. For example, overall levels of expression for each marker were generally modest compared to other studies in patients with established acute tubular necrosis [16, 28, 29]. It is known that mRNA expression of NGAL in response to ischemia is produced in the distal tubule, with much of the proximal tubular protein potentially taken up post-filtration. Thus, it is possible that the modest amounts of saline delivered may not have been enough to drive differences in severe proximal tubular injury driving KIM-1 expression or loss of NGAL, but potentially were sufficient to induce expression of NGAL in the distal tubule. Alternatively, the effects of crystalloid composition may be resulting in differences in non-renal expression of NGAL. NGAL production is not limited to the kidney and is produced in multiple cell types during systemic inflammation. Thus, it is possible that differences in urinary level of NGAL may represent differences in systemic production of NGAL in response to saline administration, which may be more evident because of the overall low levels of each biomarker.

The clinical significance of our findings is uncertain. The normal urinary concentration of NGAL is around 20 ng/m [30] and prior work has suggested cut-off value of > 150 ng/ml as diagnostic of AKI [29]. In this study, though there was a statistically significant difference in urinary concentrations of NGAL between groups, the absolute value was small and may not represent clinically relevant tubular injury.

In the original SMART trial, differences in the composite MAKE30 outcome appeared to be driven primarily by differences in renal replacement therapy and death rather than by changes in creatinine. The low overall levels of urinary biomarkers and only modest difference in NGAL between groups in the current study may support the hypothesis that differences in clinical outcomes in SMART were mediated by mechanisms other than direct tubular injury (e.g. acidosis, systemic inflammation, hemodynamic instability). Use of balanced crystalloids appeared to cause lower vasopressor requirements in both a recent randomized trial during major abdominal surgery and a secondary analysis of patients with sepsis in the SMART trial [31, 32].

Our study has several strengths. The crystalloid solution patients received was determined by study group assignment, generating similar groups and minimizing indication bias. Beginning delivery of the assigned crystalloid during fluid resuscitation in the ED ensured that patients did not receive significant volumes of the non-assigned crystalloid prior to enrollment and captured the period of highest risk for acute kidney injury [21]. Urinary concentration of NGAL and KIM-1 are accepted biomarkers of early AKI and were measured using commercially available ELISAs – facilitating reproducibility.

This study also has several limitations. In a subgroup analysis of a clinical trial, chance imbalances in patient characteristics at baseline, such as urinary biomarker level, might explain differences in observed outcomes. Many patients did not have urine available from the time of presentation to the ED, limiting the analyses attempting to adjust for baseline biomarker concentration. The small sample size precluded sufficient statistical power to detect small differences in clinical outcomes. Patients were enrolled from a single medical ICU, limiting generalizability. Treating clinicians were aware of the composition of the assigned crystalloid. The average volume of intravenous crystalloid administered was relatively small. Long-term follow-up of kidney function beyond hospital discharge was unavailable.

## Conclusions

In this ancillary analysis of a clinical trial comparing balanced crystalloids to saline among critically ill adults, balanced crystalloids were associated with modestly lower urinary concentrations of NGAL and similar urinary concentrations of KIM-1, compared with saline. Future research should examine mechanisms other than tubular injury by which use of balanced crystalloids rather than saline might affect receipt of renal replacement therapy and death.

## Supplementary Information

**Additional file 1: **Supplemental Methods. **Table S1**. Composition of the study fluids. **Table S2**. Coefficient of variation for urinary biomarkers. **Table S3**. Elixhauser comorbidity index. **Table S4**. Baseline laboratory values. **Table S5**. Volume of intravenous isotonic crystalloid by study group. **Table S6**. Volume of non-study intravenous crystalloid. **Table S7**. Laboratory values. **Table S8**. Multivariable model for urinary NGAL concentration. **Table S9**. Multivariable model for urinary KIM-1 concentration. **Table S10**. Multivariable model for Major Adverse Kidney Events within 30 days. **Table S11**. Highest stage of acute kidney injury developing after enrollment. **Figure S1**. Study group assignment during the trial. **Figure S2**. Flow of participants through the trial. **Figure S3**. Urinary biomarker levels at ED presentation and 36 h. The median (horizontal bar), interquartile range (colored box), 95% confidence interval (dashed line) for urinary NGAL and KIM-1 concentration at time of emergency department presentation (Day 0, 111 patients) and 36 h after hospital admission (Day 2, 261 patients) scaled to urinary creatinine concentration are displayed for patients in the balanced crystalloid group and saline group

## Data Availability

The datasets used and/or analyzed during the current study are available from the corresponding author on reasonable request.

## Abbreviations

SMART: Isotonic Solutions and Major Adverse Renal Events Trial
NGAL: Neutrophil gelatinase-associated lipocalin
KIM-1: Kidney injury molecule-1
AKI: Acute kidney injury
RRT: Renal replacement therapy
ICUs: Intensive care units
ED: Emergency department
KDIGO: Kidney Disease: Improving Global Outcomes
MAKE30: Major adverse kidney event within 30 days
IQR: Interquartile range
CKD: Chronic kidney disease
APACHE: Acute Physiology and Chronic Health Evaluation
CKD-EPI: Chronic Kidney Disease Epidemiology
