# Supplementary material for: Effect of balanced crystalloids versus saline on urinary biomarkers of acute kidney injury in critically ill adults
Source: BMC Nephrol. 2021 Feb 5;22:54. doi: 10.1186/s12882-021-02236-x (PMC7863046; doi:10.1186/s12882-021-02236-x)
Supplement: Supplementary file 1 — Additional file 1: Supplemental Methods. Table S1. Composition of the study fluids. Table S2. Coefficient of variation for urinary biomarkers. Table S3. Elixhauser comorbidity index. Table S4. Baseline laboratory values. Table S5. Volume of intravenous isotonic crystalloid by study group. Table S6. Volume of non-study intravenous crystalloid. Table S7. Laboratory values. Table S8. Multivariable model for urinary NGAL concentration. Table S9. Multivariable model for urinary KIM-1 concentration. Table S10. Multivariable model for Major Adverse Kidney Events within 30 days. Table S11. Highest stage of acute kidney injury developing after enrollment. Figure S1. Study group assignment during the trial. Figure S2. Flow of participants through the trial. Figure S3. Urinary biomarker levels at ED presentation and 36 h. The median (horizontal bar), interquartile range (colored box), 95% confidence interval (dashed line) for urinary NGAL and KIM-1 concentration at time of emergency department presentation (Day 0, 111 patients) and 36 h after hospital admission (Day 2, 261 patients) scaled to urinary creatinine concentration are displayed for patients in the balanced crystalloid group and saline group [file 12882_2021_2236_MOESM1_ESM.docx]

**Effect of Balanced Crystalloids versus Saline on Urinary Biomarkers of Acute Kidney Injury in Critically Ill Adults**

Supplementary Appendix

CONTENTS

[LIST OF SMART INVESTIGATORS 2](#_Toc41558225)

[SUPPLEMENTAL METHODS 3](#_Toc41558226)

[A. Definitions of Study Variables 3](#_Toc41558227)

[B. Background information on Urinary Biomarkers 6](#_Toc41558228)

[C. Analysis of effect modification (“subgroups”) 7](#_Toc41558229)

[D. Handling of Missing Baseline Creatinine 8](#_Toc41558230)

[SUPPLEMENTAL TABLES 9](#_Toc41558231)

[Table S1. Composition of the study fluids. 9](#_Toc41558232)

[Table S2. Coefficient of variation for urinary biomarkers. 10](#_Toc41558233)

[Table S3. Elixhauser comorbidity index. 11](#_Toc41558234)

[Table S4. Baseline laboratory values. 12](#_Toc41558235)

[Table S5. Volume of intravenous isotonic crystalloid by study group. 13](#_Toc41558236)

[Table S6. Volume of non-study intravenous crystalloid. 14](#_Toc41558237)

[Table S7. Laboratory values. 15](#_Toc41558238)

[Table S8. Multivariable model for urinary NGAL concentration. 16](#_Toc41558239)

[Table S9. Multivariable model for urinary KIM-1 concentration. 17](#_Toc41558240)

[Table S10. Multivariable model for Major Adverse Kidney Events within 30 days. 18](#_Toc41558241)

[Table S11. Highest stage of acute kidney injury developing after enrollment. 19](#_Toc41558242)

[SUPPLEMENTAL FIGURES 20](#_Toc41558243)

[Figure S1. Study group assignment during the trial. 20](#_Toc41558244)

[Figure S2. Flow of participants through the trial. 21](#_Toc41558245)

[Figure S3. Urinary biomarker levels at ED presentation and 36 hours. 22](#_Toc41558246)

[SUPPLEMENTAL REFERENCES 23](#_Toc41558247)

# LIST OF SMART INVESTIGATORS

Vanderbilt University Medical Center, Nashville, TN – Gordon R. Bernard, Ryan M. Brown, Jonathan D. Casey, Todd W. Rice, Matthew W. Semler (Department of Medicine, Division of Allergy, Pulmonary, and Critical Care Medicine); Christopher J. Lindsell, Li Wang (Department of Biostatistics); Jonathan P. Wanderer (Department of Biomedical Informatics and Department of Anesthesiology); Wesley H. Self (Department of Emergency Medicine); Edward D. Siew (Division of Nephrology and Hypertension, Vanderbilt Center for Kidney Disease (VCKD) and Integrated Program for AKI (VIP-AKI)); Joanna L. Stollings, (Department of Pharmaceutical Services).

# SUPPLEMENTAL METHODS

## A. Definitions of Study Variables

***Fluids***

**Intravenous fluid** – For the SMART study, intravenous fluid was defined as the intravenous administration of any formulation of any volume at any rate of 0.9% sodium chloride, lactated Ringer’s, Plasma-Lyte A®; 0.45% sodium chloride, 0.225% sodium chloride, dextrose in water, 20% or 5% human albumin solution, gelatins, dextrans, or hydroxyethyl starches. This included fluid given as a bolus, fluid given as maintenance infusions, fluid given as flushes, fluid given along with IV medications (e.g., “piggy-back”, “carrier”, “chaser”, or “driver” fluid), fluid given through pressure-bag systems, fluid given as a part of thermodilution of pulmonary artery catheters, and fluid given to maintain the patency of peripheral venous access. This did not include medication diluents or oral fluids.

**Isotonic crystalloid** – For the SMART study, the term isotonic crystalloid was used to refer to any of 0.9% sodium chloride, lactated Ringer’s, or Plasma-Lyte A®. Use of the term isotonic crystalloid was intended to distinguish these three fluids from colloid solutions and from significantly hypotonic (0.45% sodium chloride) or hypertonic (3% sodium chloride) crystalloid solutions, rather than to imply that the tonicities of 0.9% sodium chloride, lactated Ringer’s, or Plasma-Lyte A® are precisely comparable to extracellular fluid.

**Saline** – For the SMART study, 0.9% sodium chloride was referred to as saline.

**Balanced crystalloid** – For the SMART study, lactated Ringer’s or Plasma-Lyte A® were referred to as balanced crystalloids. Both lactated Ringer’s solution and Plasma-Lyte A® contain less chloride than saline (Table S1), but other differences in composition lead some clinicians to prefer one over the other for particular patients. Allowing clinicians to select either lactated Ringer’s solution or Plasma-Lyte A® when balanced crystalloids were assigned was intended to improve compliance and emulate how balanced crystalloids are used in practice (1).

***Renal Function***

**Baseline creatinine** – The value for baseline creatinine was determined in a hierarchical approach (1-3). The lowest plasma creatinine between 12 months and 24 h prior to hospital admission was used when available. If no such creatinine value was available, the lowest plasma creatinine value between 24 h prior to hospital admission and the time of ICU admission was used. If no creatinine value was available between 12 months prior to hospital admission and the time of ICU admission, a baseline creatinine value was estimated using a previously-described three-variable formula [creatinine = 0.74 − 0.2 (if female) + 0.08 (if African American) + 0.003 × age (in years)] (4).

**Acute kidney injury, stage II or greater** – Stage II or greater acute kidney injury was defined according to Kidney Disease Improving Global Outcomes (KDIGO) creatinine criteria (5). AKI present at enrollment (prevalent AKI) was defined as a first plasma creatinine measurement after enrollment at least 200% of the baseline value OR both (1) greater than 4.0 mg/dL and (2) increased at least 0.3 mg/dL from baseline. AKI developing after enrollment (incident AKI) was defined as: any creatinine value between enrollment and discharge or 30 days that was (1) increased at least 0.3 mg/dL from a preceding post-enrollment value AND (2) at least 200% of the baseline value, at least 200% of a preceding post-enrollment value, or at least 4.0 mg/dL; or urine output <0.5ml/k/h for at least 12 consecutive hours; or new receipt of RRT.

**Chronic kidney disease stage III or greater** – Chronic kidney disease stage III or greater was defined as a glomerular filtration rate less than 60 ml/min per 1.73 m^2^ as calculated by the Chronic Kidney Disease Epidemiology (CKD-EPI) Collaboration equation (6) using the patient’s baseline creatinine value.

***Outcomes***

**Major Adverse Kidney Events within 30 days (MAKE30).** The MAKE30 composite outcome (2, 3, 7, 8) was considered to have occurred when patients met one or more of the following criteria in the 30 days after ICU admission and before hospital discharge: (1) in-hospital mortality, (2) receipt of new renal replacement therapy (RRT), or (3) persistent renal dysfunction. Patients who had received RRT prior to ICU admission were ineligible to meet the new RRT or persistent renal dysfunction criteria but remained eligible to meet criteria for in-hospital mortality.

**In-hospital mortality** – In-hospital mortality was defined as death from any cause prior to hospital discharge. For calculation of secondary outcomes, in-hospital mortality was assessed 30 days after ICU admission (30-day in-hospital mortality).

**Receipt of new renal replacement therapy** – Receipt of new RRT was defined as receipt of any modality of RRT between ICU admission and the first of hospital discharge or 30 days among patients not known to have received RRT prior to ICU admission. The decision to initiate new RRT was made by treating clinicians. The study did not specify criteria defining when new RRT should be provided. At the study institution, the decision to initiate RRT for critically ill adults is made by a nephrology attending physician, in collaboration with the primary service caring for the patient. Generally, this decision takes into account the patient’s pre-illness comorbidities, acute diagnoses, severity of illness, potential indications for RRT, risk for complications from initiation of RRT, trajectory of critical illness, and prior wishes regarding supportive therapy.

**Persistent renal dysfunction** – Persistent renal dysfunction was defined as a final plasma creatinine value before hospital discharge (censored at 30 days after ICU admission) ≥ 200% of the baseline creatinine value (8).

## B. Background information on Urinary Biomarkers

Urinary concentrations of both neutrophil gelatinase-associated lipocalin (NGAL), also referred to as lipocalin-2 or oncogene 24p3, and kidney injury molecule-1 (KIM-1), also referred to as hepatitis A virus cellular receptor 1 or T-cell immunoglobulin and mucin domain 1, have been demonstrated to rise rapidly in the urine of humans and rodents in response to renal tubular injury (9-12). In a rat model of fluid-resuscitated sepsis, urine NGAL concentration in the urine was found to increase 3 hours after induction of sepsis and peak from 24 to 48 hours. In the same study, urine KIM-1 concentration peaked at 6 hours and returned to baseline by 72 hours (12).

The normal urine concentration of NGAL is ~20 ng/mL and prior work has suggested a cut-off value of >150 ng/ml as diagnostic of AKI (13). In the setting of acute renal failure, human urinary NGAL levels have been found to increase 25-fold, with only slightly elevated urinary NGAL levels being found in patients with chronic renal failure (14).

KIM-1 is rarely detectable in the urine of individuals without acute or chronic renal disease (10, 11). A study in humans found a one-unit increase in normalized KIM-1 to be strongly associated with a diagnosis of acute tubular necrosis, with a mean KIM-1 concentration (normalized to urine creatinine) of 2.92 ng/mg in patients diagnosed with ischemic acute tubular necrosis (15).

## C. Analysis of effect modification (“subgroups”)

Using proportional odds modeling, we examined the interaction between crystalloid assignment and the following pre-specified baseline variables with respect to the primary outcome of day 2 urinary biomarker levels in the intention-to-treat population:

a. Prevalent AKI (*yes, no*)

b. Measured baseline creatinine (*yes, no*)

c. Sepsis or septic shock (*yes, no*)

d. APACHE score (*>10, <10*)

## D. Handling of Missing Baseline Creatinine

For patients without a measured plasma creatinine between 12 months prior to hospital admission and enrollment, baseline creatinine value for the primary analysis was estimated using a previously-described three-variable formula [creatinine = 0.74 − 0.2 (if female) + 0.08 (if African American) + 0.003 × age (in years)]. (4)

# SUPPLEMENTAL TABLES

## Table S1. Composition of the study fluids.

|  | **Sodium** | **Potassium** | **Calcium** | **Magnesium** | **Chloride** | **Acetate** | **Lactate** | **Gluconate** | **Osmolarity** |
| --- | --- | --- | --- | --- | --- | --- | --- | --- | --- |
| Plasma | 135–145 | 4.5–5.0 | 2.2–2.6 | 0.8–1.0 | 94–111 |  | 1–2 |  | 275–295 |
| 0.9% saline | 154 |  |  |  | 154 |  |  |  | 308 |
| Lactated Ringer’s | 130 | 4.0 | 2.7 |  | 109 |  | 28 |  | 273 |
| Plasma-Lyte A® | 140 | 5.0 |  | 3.0 | 98 | 27 |  | 23 | 294 |

All values are in mEq/L except calculated osmolarity, which is in mOsm/L. 0.9% saline is “Sodium Chloride Injection, USP”, lactated Ringer’s is “lactated Ringer’s Injection, USP”, and Plasma-Lyte A® is “Multiple Electrolyte Injection, Type 1, USP”, all from Baxter Healthcare Corporation in Deerfield, IL, USA.

## Table S2. Coefficient of variation for urinary biomarkers.

|  | **Creatinine (mg/dl)** | **KIM-1 (ng/ml)** | **NGAL (ng/ml)** |
| --- | --- | --- | --- |
| Day 0 (ED presentation) | 3.24 | 5.54 | 8.07 |
| Day 2 (36 ± 12 hours after hospital admission) | 2.04 | 3.53 | 6.74 |

Coefficient of variation presented as an average percentage. ED is emergency department.

## Table S3. Elixhauser comorbidity index.

|  | **Balanced** | **Saline** |
| --- | --- | --- |
| **Comorbidity, No. (%)*** | **(n = 131)** | **(n = 130)** |
| Congestive heart failure | 16 (12%) | 12 (9%) |
| Cardiac arrhythmias | 18 (14%) | 21 (16%) |
| Coronary artery disease | 31 (24%) | 24 (18%) |
| Hypertension | 79 (60%) | 69 (53%) |
| Diabetes | 52 (40%) | 49 (38%) |
| Cerebrovascular accident | 12 (9%) | 15 (12%) |
| Dementia | 7 (5%) | 6 (5%) |
| Chronic respiratory failure | 40 (31%) | 39 (30%) |
| Solid tumor | 24 (18%) | 32 (25%) |
| Leukemia or lymphoma | 7 (5%) | 7 (5%) |
| Cirrhosis | 19 (15%) | 9 (7%) |
| Peptic ulcer disease | 9 (7%) | 10 (8%) |
| Chronic kidney disease† | 18 (14%) | 14 (11%) |
| Human immunodeficiency virus | 3 (2%) | 5 (4%) |
| Immunosuppression | 30 (23%) | 35 (27%) |

*The Elixhauser Comorbidity Index is a method for measuring patient comorbidity based on the International Classification of Diseases (ICD) diagnosis codes (ICD-9-CM and ICD-10) found in administrative data (16, 17). There were no significant differences in baseline comorbidities between the two study groups.

†Stage 3 or greater as determined by baseline creatinine and defined as a glomerular filtration rate less than 60 ml/min per 1.73 m^2^ as calculated by the Chronic Kidney Disease Epidemiology (CKD-EPI) Collaboration equation using the patient’s baseline creatinine value.

## Table S4. Baseline laboratory values.

|  |  | **Balanced** | **Saline** |
| --- | --- | --- | --- |
| **Most recent value in 12 months prior to hospitalization*** | **n** | **(n = 131)** | **(n = 130)** |
| Plasma creatinine, mg/dL | 178 | 0.89 [0.74-1.20] | 0.90 [0.72-1.16] |
|  |  |  |  |
| **First value between hospitalization and ICU admission†** |  |  |  |
| Plasma sodium, mmol/L | 261 | 137 [134-140] | 137 [133-139] |
| Plasma potassium, mmol/L | 261 | 4.1 [3.7-4.9] | 4.2 [3.7-4.7] |
| Plasma chloride, mmol/L | 261 | 101 [98-106] | 102 [98-106] |
| Plasma bicarbonate, mmol/L | 261 | 22 [18-25] | 22 [18-24] |
| Plasma blood urea nitrogen, mg/dL | 261 | 22 [14-32] | 19 [12-29] |
| Plasma creatinine, mg/dL | 261 | 1.1 [0.8-1.7] | 1.1 [0.8-17] |
|  |  |  |  |
| **Baseline creatinine‡ – mg/dL** | 261 | 0.77 [0.67-0.90] | 0.78 [0.65-0.90] |
| Among patients with a value in 12 months prior§ - median [IQR], mg/dL | 178 | 0.79 [0.65-0.92] | 0.72 [0.60-0.95] |

Data are presented as median [25th percentile – 75th percentile]

* Most recent value in 12 months prior to hospitalization is defined as the most recent value in the time period between one year prior to hospital admission and 24 hours prior to hospital admission

† First value between hospitalization and ICU admission is defined as first value obtained after presentation to the ED.

‡ Baseline creatinine for the study is defined as the lowest plasma creatinine measured in the 12 months prior to hospitalization if available, otherwise the lowest plasma creatinine measured between hospitalization and ICU admission; using the estimated creatinine only for patients without an available plasma creatinine between 12 months prior to hospitalization and the time of ICU admission.

§ Lowest creatinine in the 12 months prior to hospitalization is defined as the lowest available plasma creatinine between 12 months and 24 hours prior to hospital admission.

## Table S5. Volume of intravenous isotonic crystalloid by study group.

|  | **Balanced Crystalloid** | **Saline** |  |
| --- | --- | --- | --- |
|  | **(n = 131)** | **(n = 130)** | ***P* value** |
| **0.9% sodium chloride, median [IQR]; mean ± SD, mL** |  |  |  |
| In the 24 hours prior to ICU admission | 0 [0 – 0]; 307 ± 787 | 1000 [0 – 2000]; 1372 ± 1654 | <0.001 |
| Day 0 24-hour fluid receipt | 0 [0 – 0]; 67 ± 243 | 0 [0 – 1009]; 710 ± 1097 | <0.001 |
| Day 1 24-hour fluid receipt | 0 [0 – 0]; 58 ± 362 | 120 [0 – 1000]; 644 ± 1030 | <0.001 |
| Day 2 24-hour fluid receipt | 0 [0 – 0]; 27 ± 263 | 0 [0 – 110]; 288 ± 735 | <0.001 |
| Cumulative volume from ICU admission through day 2 | 0 [0 – 0]; 153 ± 618 | 1032 [0 – 2776]; 1641 ± 1973 | <0.001 |
|  |  |  |  |
| **Balanced crystalloid, median [IQR]; mean ± SD, mL** |  |  |  |
| In the 24 hours prior to ICU admission | 500 [0 – 2000]; 991 ± 1189 | 0 [0 – 0]; 13 ± 98 | <0.001 |
| Day 0 24-hour fluid receipt | 500 [0 – 1750]; 995 ± 1262 | 0 [0 –0]; 67 ± 487 | <0.001 |
| Day 1 24-hour fluid receipt | 30 [0 – 1000]; 550 ± 902 | 0 [0 – 0]; 90 ± 541 | <0.001 |
| Day 2 24-hour fluid receipt | 0 [0 – 85]; 367 ± 992 | 0 [0 – 0]; 99 ± 571 | <0.001 |
| Cumulative volume from ICU admission through day 2 | 1070 [5 – 3124]; 1911 ± 2277 | 0 [0 – 0]; 256 ± 1499 | <0.001 |
|  |  |  |  |
| **Lactated Ringer’s, median [IQR]; mean ± SD, mL** |  |  |  |
| In the 24 hours prior to ICU admission | 500 [0 – 1750]; 964 ± 1147 | 0 [0 – 0]; 12 ± 98 | <0.001 |
| Day 0 24-hour fluid receipt | 500 [0 – 1652]; 972 ± 1248 | 0 [0 – 0]; 64 ± 486 | <0.001 |
| Day 1 24-hour fluid receipt | 30 [0 – 1000]; 550 ± 902 | 0 [0 – 0]; 81 ± 536 | <0.001 |
| Day 2 24-hour fluid receipt | 0 [0 – 60]; 359 ± 991 | 0 [0 – 0]; 87 ± 562 | <0.001 |
| Cumulative volume from ICU admission through day 2 | 1070 [0 – 3124]; 1881 ± 2258 | 0 [0 – 0]; 232 ± 1490 | <0.001 |
|  |  |  |  |
| **Plasma-Lyte A®, median [IQR]; mean ± SD, mL** |  |  |  |
| In the 24 hours prior to ICU admission | 0 [0 – 0]; 27 ± 199 | 0 [0 – 0]; 1 ± 13 | 0.31 |
| Day 0 24-hour fluid receipt | 0 [0 – 0]; 23 ± 195 | 0 [0 – 0]; 4 ± 44 | 0.56 |
| Day 1 24-hour fluid receipt | 0 [0 – 0]; 0 ± 0 | 0 [0 – 0]; 9 ± 81 | 0.15 |
| Day 2 24-hour fluid receipt | 0 [0 – 0]; 8 ± 87 | 0 [0 – 0]; 12 ± 108 | 0.56 |
| Cumulative volume from ICU admission through day 2 | 0 [0 – 0]; 31 ± 213 | 0 [0 – 0]; 24 ± 191 | 0.71 |

Fluid in the 24 hours prior to ICU admission includes fluid ordered in the emergency department but does not include fluid ordered prior to arrival to the study institution. Cumulative volume of fluid ordered from ICU admission through day 2 includes fluid ordered both in the intensive care unit (ICU) and after transfer out of the ICU. Balanced crystalloid includes lactated Ringer’s and Plasma-Lyte A®.

## Table S6. Volume of non-study intravenous crystalloid.

|  | **Balanced Crystalloid** | **Saline** |  |
| --- | --- | --- | --- |
|  | **(n = 131)** | **(n = 130)** | ***P* value** |
| **Hypotonic crystalloid, median [IQR]; mean ± SD, mL** |  |  |  |
| In the 24 hours prior to ICU admission | 0 [0 – 0]; 11 ± 63 | 0 [0 – 0]; 1 ± 7 | 0.10 |
| Day 0 24-hour fluid receipt | 0 [0 – 0]; 39 ± 180 | 0 [0 – 0]; 75 ± 345 | 0.93 |
| Day 1 24-hour fluid receipt | 0 [0 – 0]; 55 ± 288 | 0 [0 – 0]; 48 ± 234 | 0.99 |
| Day 2 24-hour fluid receipt | 0 [0 – 0]; 0 ± 0 | 0 [0 – 0]; 13 ± 102 | 0.08 |
| Cumulative volume from ICU admission through day 2 | 0 [0 – 0]; 94 ± 397 | 0 [0 – 0]; 136 ± 525 | 0.93 |

Hypotonic Crystalloid includes 0.45% sodium chloride, 0.225% sodium chloride, and dextrose in water.

## Table S7. Laboratory values.

|  | **Balanced** | **Saline** |  |
| --- | --- | --- | --- |
| **Laboratory value** | **(n = 131)** | **(n = 130)** | ***P* value** |
| Plasma sodium, mmol/L |  |  |  |
| Day 0 | 137 [134 – 140] | 137 [133 – 139] | 0.22 |
| Day 1 | 138 [135 – 140] | 137 [134 – 139] | 0.23 |
| Day 2 | 138 [136 – 140] | 137 [135 – 139] | 0.083 |
| Day 3 | 138 [135 – 140] | 138 [135 – 140] | 0.64 |
|  |  |  |  |
| Plasma potassium, mmol/L |  |  |  |
| Day 0 | 4.1 [3.7 – 4.9] | 4.2 [3.7 – 4.7] | 0.85 |
| Day 1 | 4.0 [3.6 – 4.5] | 4.1 [3.7 – 4.5] | 0.44 |
| Day 2 | 3.8 [3.6 – 4.3] | 3.9 [3.5 – 4.3] | 0.78 |
| Day 3 | 3.8 [3.5 – 4.2] | 3.8 [3.4 – 4.1] | 0.81 |
|  |  |  |  |
| Plasma chloride, mmol/L |  |  |  |
| Day 0 | 101 [98 – 106] | 102 [98 – 106] | 0.53 |
| Day 1 | 104 [101 – 108] | 107 [103 – 100] | 0.002 |
| Day 2 | 104 [100 – 107] | 107 [102 – 110] | <0.001 |
| Day 3 | 103 [100 – 107] | 105 [102 – 108] | 0.01 |
|  |  |  |  |
| Plasma bicarbonate, mmol/L |  |  |  |
| Day 0 | 22 [18 – 25] | 22 [18 – 24] | 0.73 |
| Day 1 | 23 [19 – 26] | 21 [18 – 23] | <0.001 |
| Day 2 | 24 [21 – 26] | 22 [19 – 24] | <0.001 |
| Day 3 | 24 [22 – 27] | 23 [ 20 – 26] | 0.026 |

Data are presented as median [25th percentile – 75th percentile]

## Table S8. Multivariable model for urinary NGAL concentration.

| **Variable** | **Odds Ratio** | **95% CI** | ***P* value** |
| --- | --- | --- | --- |
| Study Group (Balanced crystalloids : Saline) | 0.47 | 0.30 – 0.72 | <0.001 |
| Age, years | 1.22 | 0.89 – 1.66 | 0.22 |
| Sex (Female : Male) | 2.15 | 1.38 – 3.34 | <0.001 |
| Race (Non-White : White) | 1.18 | 0.65 – 2.14 | 0.59 |
| Mechanical Ventilation (Yes : No) | 0.85 | 0.46 – 1.57 | 0.60 |
| Vasopressor Receipt (Yes : No) | 1.44 | 0.59 – 3.55 | 0.43 |
| Sepsis or Septic Shock (Yes : No) | 3.33 | 1.88 – 5.88 | <0.001 |

This table presents the results of an intention-to-treat comparison of the co-primary outcome of urinary NGAL level 36 ± 12 hours after hospital admission between the balanced crystalloid and saline groups using a proportional odds model with covariates for group assignment, age, sex, race, mechanical ventilation, vasopressor receipt, and diagnosis of sepsis. In this analysis of a non-parametrically distributed continuous outcome using a proportional odds model, an odds ratio below 1.0 corresponds to a probability of lower NGAL concentration in the balanced crystalloid group compared with the saline group.

## Table S9. Multivariable model for urinary KIM-1 concentration.

| **Variable** | **Odds Ratio** | **95% CI** | ***P* value** |
| --- | --- | --- | --- |
| Study Group (Balanced crystalloids : Saline) | 1.25 | 0.81 – 1.91 | 0.31 |
| Age, years | 1.42 | 1.04 – 1.94 | 0.03 |
| Sex (Female : Male) | 0.89 | 0.58 – 1.37 | 0.60 |
| Race (Non-White : White) | 0.45 | 0.25 – 0.81 | 0.01 |
| Mechanical Ventilation (Yes : No) | 0.78 | 0.42 – 1.45 | 0.43 |
| Vasopressor Receipt (Yes : No) | 0.89 | 0.37 – 2.13 | 0.80 |
| Sepsis or Septic Shock (Yes : No) | 1.96 | 1.12 – 3.33 | 0.02 |

This table presents the results of an intention-to-treat comparison of the co-primary outcome of urinary KIM-1 level 36 ± 12 hours after hospital admission between the balanced crystalloid and saline groups using a proportional odds model with covariates for group assignment, age, sex, race, mechanical ventilation, vasopressor receipt, and diagnosis of sepsis. In this analysis of a non-parametrically distributed continuous outcome using a proportional odds model, an odds ratio above 1.0 corresponds to a probability of higher KIM-1 concentration in the balanced crystalloid group compared with the saline group.

## Table S10. Multivariable model for Major Adverse Kidney Events within 30 days.

| **Variable** | **Odds Ratio** | **95% CI** | ***P* value** |
| --- | --- | --- | --- |
| Study Group (Balanced crystalloids : Saline) | 0.90 | 0.39 – 2.05 | 0.80 |
| Age, years | 1.02 | 0.99 – 1.04 | 0.16 |
| Sex (Male : Female) | 1.13 | 0.5 – 2.53 | 0.78 |
| Race (Non-White : White) | 0.40 | 0.15 – 1.06 | 0.07 |
| Mechanical Ventilation (Yes : No) | 1.44 | 0.42 – 4.95 | 0.56 |
| Vasopressor Receipt (Yes : No) | 0.19 | 0.04 – 0.81 | 0.015 |
| Sepsis or Septic Shock (Yes : No) | 1.89 | 0.54 – 6.58 | 0.27 |

This table presents the results of an intention-to-treat comparison of the primary outcome of Major Adverse Kidney Events within 30 days (MAKE30) between the balanced crystalloid and saline groups using a logistic regression model with covariates of group assignment, age, sex, race, mechanical ventilation, vasopressor receipt, and diagnosis of sepsis. All components of the MAKE30 composite outcome were censored at the time of hospital discharge.

## Table S11. Highest stage of acute kidney injury developing after enrollment.

|  | **Balanced**  **(n=131)** | **Saline**  **(n=130)** | ***P* value** |
| --- | --- | --- | --- |
| **Highest stage of incident AKI by KDIGO criteria, No (%)** |  |  | 0.18 |
| None | 33 (25) | 47 (37) |  |
| Stage I | 47 (36) | 33 (26) |  |
| Stage II | 28 (22) | 27 (21) |  |
| Stage III | 22 (17) | 21 (16) |  |

In this *post-hoc* analysis, the highest stage of acute kidney injury (AKI) developing between enrollment and the first of hospital discharge or 30 days is compared between study groups. Incident AKI is defined using Kidney Disease Improving Global Outcomes (KDIGO) creatinine criteria (5) or study day urine output as follows:

- Stage I AKI is defined as a plasma creatinine value that is increased ≥ 0.3 mg/dL from a prior post-enrollment value and either 1.5-1.9 times greater than baseline or 1.5-1.9 times greater than the lowest prior on-study value; or urine output <0.5ml/kg/h for at least 6 consecutive hours;
- Stage II AKI is defined as a creatinine value that is increased ≥ 0.3 mg/dL from a prior post-enrollment value and either 2.0-2.9 times greater than baseline or 2.0-2.9 times greater than the lowest prior post-enrollment value; or urine output <0.5ml/kg/h for at least 12 consecutive hours; and
- Stage III AKI is defined as receipt of new RRT or a creatinine value that is increased ≥ 0.3 mg/dL from a prior post-enrollment value and either ≥ 3.0 times greater than baseline, ≥ 3.0 times greater than the lowest prior post-enrollment value, or ≥ 4.0 mg/dL; or urine output <0.3mg/kh/h for at least 24 hours or anuria for 12 or more hours; or new RRT.

# SUPPLEMENTAL FIGURES

## Figure S1. Study group assignment during the trial.

**
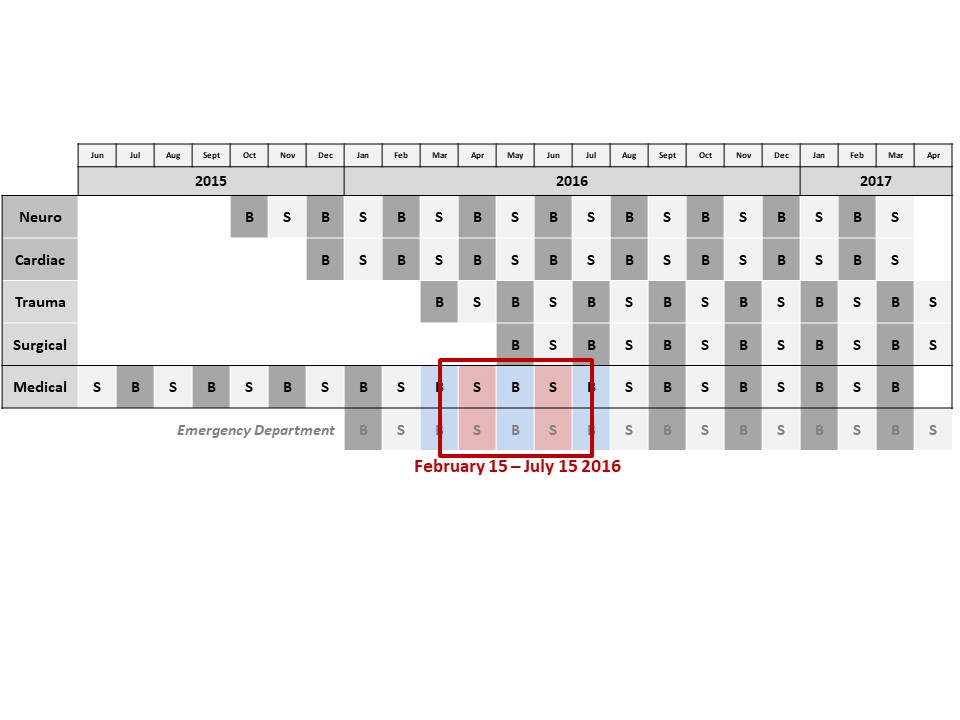
**

The cohort for the current study included all patients co-enrolled in SMART and the Vanderbilt University Emergency Medicine Biomarkers Study who were admitted from the emergency department and remained in the medical intensive care unit at 36 hours after admission between February 15 2016 and July 15 2016.

## Figure S2. Flow of participants through the trial.


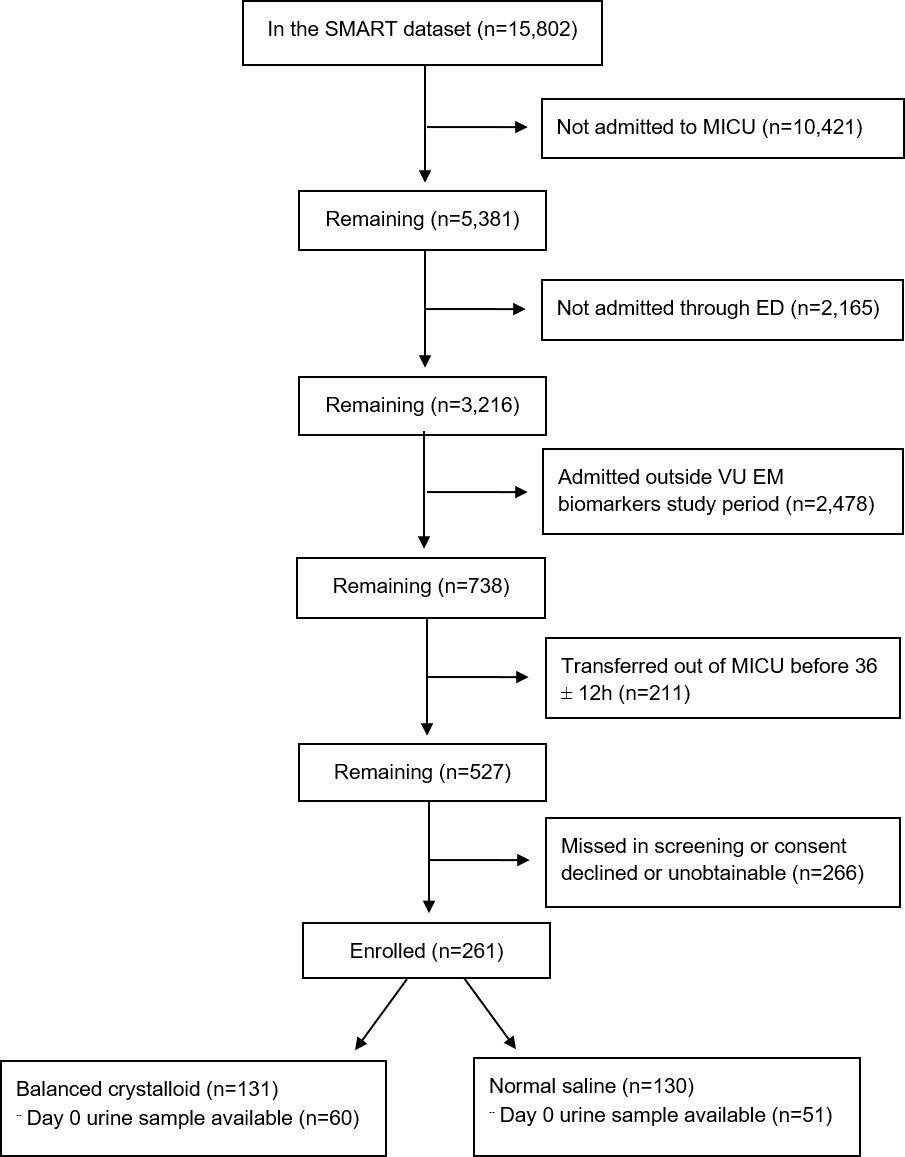


## Figure S3. Urinary biomarker levels at ED presentation and 36 hours.

The median (horizontal bar), interquartile range (colored box), 95% confidence interval (dashed line) for urinary NGAL and KIM-1 concentration at time of emergency department presentation (Day 0, 111 patients) and 36 hours after hospital admission (Day 2, 261 patients) scaled to urinary creatinine concentration are displayed for patients in the balanced crystalloid group and saline group.

# SUPPLEMENTAL REFERENCES

1. Semler MW, Self WH, Wang L, et al. Balanced crystalloids versus saline in the intensive care unit: study protocol for a cluster-randomized, multiple-crossover trial. Trials 2017;18(1):129.

2. Semler MW, Rice TW, Shaw AD, et al. Identification of Major Adverse Kidney Events Within the Electronic Health Record. J Med Syst 2016;40(7):167.

3. Semler MW, Wanderer JP, Ehrenfeld JM, et al. Balanced Crystalloids versus Saline in the Intensive Care Unit. The SALT Randomized Trial. Am J Respir Crit Care Med 2017;195(10):1362–72.

4. Závada J, Hoste E, Cartin-Ceba R, et al. A comparison of three methods to estimate baseline creatinine for RIFLE classification. Nephrol Dial Transplant Off Publ Eur Dial Transpl Assoc - Eur Ren Assoc 2010;25(12):3911–8.

5. Kidney Disease: Improving Global Outcomes (KDIGO) Acute Kidney Injury Work Group. KDIGO Clinical Practice Guideline for Acute Kidney Injury. Kidney inter 2012;2(Suppl):8.

6. Levey AS, Stevens LA, Schmid CH, et al. A new equation to estimate glomerular filtration rate. Ann Intern Med 2009;150(9):604–12.

7. Palevsky PM, Molitoris BA, Okusa MD, et al. Design of clinical trials in acute kidney injury: report from an NIDDK workshop on trial methodology. Clin J Am Soc Nephrol CJASN 2012;7(5):844–50.

8. Kashani K, Al-Khafaji A, Ardiles T, et al. Discovery and validation of cell cycle arrest biomarkers in human acute kidney injury. Crit Care Lond Engl 2013;17(1):R25.

9. Schmidt-Ott KM, Mori K, Li JY, et al. Dual action of neutrophil gelatinase-associated lipocalin. J Am Soc Nephrol JASN 2007;18(2):407–13.

10. Bonventre JV. Kidney injury molecule-1 (KIM-1): a urinary biomarker and much more. Nephrol Dial Transplant Off Publ Eur Dial Transpl Assoc - Eur Ren Assoc 2009;24(11):3265–8.

11. Song J, Yu J, Prayogo GW, et al. Understanding kidney injury molecule 1: a novel immune factor in kidney pathophysiology. Am J Transl Res 2019;11(3):1219–29.

12. Arulkumaran N, Sixma ML, Jentho E, et al. Sequential Analysis of a Panel of Biomarkers and Pathologic Findings in a Resuscitated Rat Model of Sepsis and Recovery. Crit Care Med 2017;45(8):e821–30.

13. Haase-Fielitz A, Haase M, Devarajan P. Neutrophil gelatinase-associated lipocalin as a biomarker of acute kidney injury: a critical evaluation of current status. Ann Clin Biochem 2014;51(Pt 3):335–51.

14. Mori K, Lee HT, Rapoport D, et al. Endocytic delivery of lipocalin-siderophore-iron complex rescues the kidney from ischemia-reperfusion injury. J Clin Invest 2005;115(3):610–21.

15. Han WK, Bailly V, Abichandani R, Thadhani R, Bonventre JV. Kidney Injury Molecule-1 (KIM-1): a novel biomarker for human renal proximal tubule injury. Kidney Int 2002;62(1):237–44.

16. Elixhauser A, Steiner C, Harris DR, Coffey RM. Comorbidity measures for use with administrative data. Med Care 1998;36(1):8–27.

17. Quan H, Sundararajan V, Halfon P, et al. Coding algorithms for defining comorbidities in ICD-9-CM and ICD-10 administrative data. Med Care 2005;43(11):1130–9.
